# Molecular screening with liquid biopsy for anti-EGFR retreatment in metastatic colorectal cancer: preliminary data from the randomized phase 2 PARERE trial

**DOI:** 10.3389/fonc.2023.1307545

**Published:** 2024-02-09

**Authors:** Marco Maria Germani, Guglielmo Vetere, Mirella Giordano, Paolo Ciracì, Iolanda Capone, Elena Tamborini, Elena Conca, Adele Busico, Filippo Pietrantonio, Vittoria Matilde Piva, Alessandra Boccaccino, Francesca Simionato, Martina Bortolot, Paolo Manca, Sara Lonardi, Veronica Conca, Beatrice Borelli, Martina Carullo, Marzia Del Re, Gabriella Fontanini, Daniele Rossini, Chiara Cremolini

**Affiliations:** ^1^ Unit of Medical Oncology 2, Azienda Ospedaliera Universitaria Pisana, Pisa, Italy; ^2^ Department of Translational Research and New Technologies in Medicine and Surgery, University of Pisa, Pisa, Italy; ^3^ Molecular Pathology Laboratory, Department of Pathology, Fondazione Istituto di Ricovero e Cura a Carattere Scientifico (IRCCS) Istituto Nazionale Dei Tumori, Milan, Italy; ^4^ Department of Medical Oncology, Fondazione Istituto di Ricovero e Cura a Carattere Scientifico (IRCCS) Istituto Nazionale dei Tumori, Milan, Italy; ^5^ Oncology Unit 1, Veneto Institute of Oncology - Istituto di Ricovero e Cura a Carattere Scientifico (IRCCS), Padua, Italy; ^6^ Department of Surgical, Oncological, and Gastroenterological Sciences, University of Padua, Padua, Italy; ^7^ Department of Oncology, San Bortolo General Hospital, Vicenza, Italy; ^8^ Department of Medicine (DAME), University of Udine, Udine, Italy; ^9^ Department of Oncology, Azienda Sanitaria Universitaria Friuli Centrale (ASUFC), Udine, Italy; ^10^ Oncology Unit 3, Veneto Institute of Oncology - Istituto di Ricovero e Cura a Carattere Scientifico (IRCCS), Padua, Italy; ^11^ Unit of Clinical Pharmacology and Pharmacogenetics, Department of Clinical and Experimental Medicine, University of Pisa, Pisa, Italy; ^12^ Department of Surgical, Medical, Molecular Pathology and Critical Area, University of Pisa, Pisa, Italy; ^13^ Section of Clinical Pharmacology and Oncology, Department of Health Sciences, University of Florence, Florence, Italy

**Keywords:** mCRC, anti-EGFR, retreatment, ctDNA, liquid biopsy, next generation sequencing

## Abstract

**Background:**

Retreatment with anti-EGFR monoclonal antibodies is a promising strategy in patients with *RAS/BRAF* wild-type (wt) metastatic colorectal cancer (mCRC) who achieved benefit from previous anti-EGFR exposure upon exclusion of mutations in *RAS/BRAF* genes according to circulating tumor DNA (ctDNA) analysis by means of liquid biopsy (LB). This treatment approach is now being investigated in the randomized phase II trial PARERE (NCT04787341). We here present preliminary findings of molecular screening.

**Methods:**

Patients with *RAS/BRAF*V600E wt mCRC according to tissue genotyping who benefited from previous anti-EGFR-based treatment (fluoropyrimidines, oxaliplatin, irinotecan, and antiangiogenics) and then experienced disease progression to EGFR targeting were eligible for screening in the PARERE trial. The next-generation sequencing (NGS) panel Oncomine™ was employed for ctDNA testing.

**Results:**

A total of 218 patients underwent LB, and ctDNA sequencing was successful in 201 of them (92%). *RAS/BRAF*V600E mutations were found in 68 (34%) patients and were mainly subclonal (median variant allele fraction [VAF] for *KRAS*, *NRAS*, and *BRAF* mutant clones: 0.52%, 0.62%, and 0.12%, respectively; *p* = 0.01), with *KRAS*Q61H being the most frequently detected (31%). Anti-EGFR-free intervals did not predict ctDNA molecular status (*p* = 0.12). Among the 133 patients with *RAS/BRAF*V600E wt tumors according to LB, 40 (30%) harbored a mutation in at least another gene potentially implied in anti-EGFR resistance, mainly with subclonal expression (median VAF, 0.56%). In detail, alterations in *PIK3CA*, *FBXW7*, *GNAS*, *MAP2K*, *ERBB2*, *BRAF* (class I and II non-BRAFV600E), *SMAD*, *EGFR*, *AKT1*, and *CTNNB1* occurred in 13%, 8%, 7%, 3%, 2%, 2%, 1%, 1%, 1%, and 1% cases, respectively. Co-mutations were detected in 13 (33%) out of 40 patients.

**Conclusions:**

This is the largest prospective cohort of mCRC patients screened with LB for anti-EGFR retreatment in a randomized study. ctDNA genotyping reveals that at least one out of three patients candidate for retreatment should be excluded from this therapy, and other potential drivers of anti-EGFR resistance are found in approximately one out of three patients with *RAS/BRAF*V600E wt ctDNA.

## 1 Introduction

Retreatment with monoclonal antibodies targeting the anti-epidermal growth factor receptors (anti-EGFRs) is a promising approach for *RAS/BRAF* wild-type (wt) metastatic colorectal cancer (mCRC) patients who developed acquired resistance to previous anti-EGFR exposure (1–6). However, the overall response rates (ORRs) observed in initial single-arm phase II trials range between 0% and 21%, suggesting the need for a more accurate patient selection (2–4, 6).

To this end, circulating tumor DNA (ctDNA) sequencing in liquid biopsy (LB) is regarded as the preferred approach to fine-tune the identification of patients candidate for anti-EGFR retreatment, as reported by *post-hoc* translational analyses of single-arm phase II studies, showing no benefit in patients with *RAS/BRAF* mutant ctDNA, and the recent prospective ctDNA-guided CHRONOS trial, reporting an ORR of 30% in patients with *RAS/BRAF* wt ctDNA (1–3, 6). Similarly, a multi-arm non-comparative phase 2 trial by Parseghian et al. showed an ORR of 20% to anti-EGFR retreatment in patients with no acquired mutations in *RAS/BRAF/MAP2K/EGFR*, and no responses to anti-EGFR retreatment in patients harboring one of these mutations in their ctDNA (4). Conversely, the evaluation of clinical biomarkers as surrogates of *RAS/BRAF* mutational status led to conflicting results (7), thus endorsing the prospective adoption of molecular screening with LB in recent studies on anti-EGFR retreatment and in the real-world setting where anti-EGFR retreatment was proven feasible and active (ORR, 25%) in patients with no *RAS* or *BRAF* mutations in their ctDNA (5, 8, 9).

These data consistently suggest that while up to one-third of patients may benefit from anti-EGFR retreatment in the case of *RAS/BRAF* wt ctDNA, approximately two-thirds of them still do not respond to anti-EGFR re-exposure, including one-third of rapid progressors (1, 7). Therefore, *RAS* and *BRAF* genotyping alone does not allow to catch the wide complexity of escape mechanisms to EGFR blockade, so the adoption of broader genomic panels (i.e., next-generation sequencing [NGS] assays) may further improve molecular selection in potential candidates for anti-EGFR retreatment. In this research framework, we are now conducting the phase II PARERE trial (NCT04787341) randomizing patients previously exposed to first-line anti-EGFR-containing regimens and not harboring *RAS/BRAF* mutations in their ctDNA to receive panitumumab retreatment followed by regorafenib versus the reverse strategy. The NGS Oncomine™ (10) assay, including 14 genes implied in mCRC progression and anti-EGFR resistance, is used as a screening tool. Here, we present preliminary molecular findings of the screening phase.

## 2 Materials and methods

### 2.1 Patient population

*RAS/BRAF* wild-type mCRC patients enrolled in the molecular screening phase between December 2020 and January 2023 of the randomized phase II PARERE study (NCT04787341) were included. Candidates had to meet the following criteria: diagnosis of unresectable mCRC previously treated with fluoropyrimidines, oxaliplatin, irinotecan, and an antiangiogenic agent (bevacizumab or aflibercept); *KRAS/NRAS* (codons 12, 13, 59, 61, 117, and 146) and *BRAF*V600E wt status on primary tumor and/or metastasis; a partial response or stable disease ≥6 months during a previous anti-EGFR-based first-line treatment; ≥4 months interval between the last anti-EGFR administration and LB.

### 2.2 Sequencing methodology

NGS analysis with Oncomine™ Colon cfDNA Assay was performed at Fondazione IRCSS - Istituto Nazionale dei Tumori di Milano, Milan, Italy. This assay detects frequently mutated single-nucleotide variants (SNVs) and short indels in colon/gastro-intestinal cancers, covering 14 genes with >240 hotspots [Protein Kinase B (*AKT1*), B-raf Murine Sarcoma Viral Oncogene Homologue B (*BRAF*), Catenin Beta-1 (*CTNNB1*), EGFR, Erb-B2 Receptor Tyrosine Kinase 2 (*ERBB2*), F-box/WD Repeat Containing Protein 7 (*FBXW7*), Guanine Nucleotide Binding Protein (*GNAS*), Kirsten Rat Sarcoma Virus (*KRAS*), Mitogen-activated Protein Kinase Kinase (*MAP2K1*), Neuroblastoma Ras Viral Oncogene Homologue (*NRAS*), Phosphatidylinositol 4,5-Bisphosphate 3-Kinase Catalytic Subunit Alpha Isoform (*PIK3CA*), Mothers Against Decapentaplegic (*SMAD4*), Tumor Protein P53 (*TP53*), and Adenomatous Polyposis Coli (*APC*)] with a flexible limit of detection (LOD) of 0.1%–5% that varies with the cfDNA input (1–20 ng). Briefly, sequencing was performed *via* the use of a tag on plasma samples of circulating free DNA: after attaching a unique molecular tag to the gene-specific primers, the amplified products were grouped into families harboring the same tags. Families containing the same mutant variant were called with optimized Variant Caller settings for the Oncology-Liquid Biopsy application. Families that contained random errors were identified and removed from variant calling. The same test was performed in all samples from patients undergoing molecular screening for the PARERE study.

### 2.3 Statistical analysis

Patients’ characteristics were described as counts and percentages in the case of categorical variables and as ranges or 95% confidence intervals in the case of continuous variables. Comparisons of the clinical characteristics of the *RAS/BRAF* wt and mutant groups were performed with the Mann–Whitney, chi-squared, and Fisher’s exact tests, where appropriate. ORR was defined as the ratio between the number of patients achieving at least a partial response, according to Response Evaluation Criteria in Solid Tumors (RECIST) 1.1 criteria (11), and the number of patients who underwent objective radiological assessment at first anti-EGFR exposure. Progression-free survival (PFS) was defined as the interval from the first anti-EGFR exposure to disease progression. LODs of the Oncomine™ panel for *KRAS*, *NRAS*, and *BRAF* mutant clones were compared with the Kruskal–Wallis test and Dunn’s multiple comparison test. Data analysis and visualization were performed with RStudio version 2022.07.02 and Prisma version 4.7.

## 3 Results

**Figure 1:**
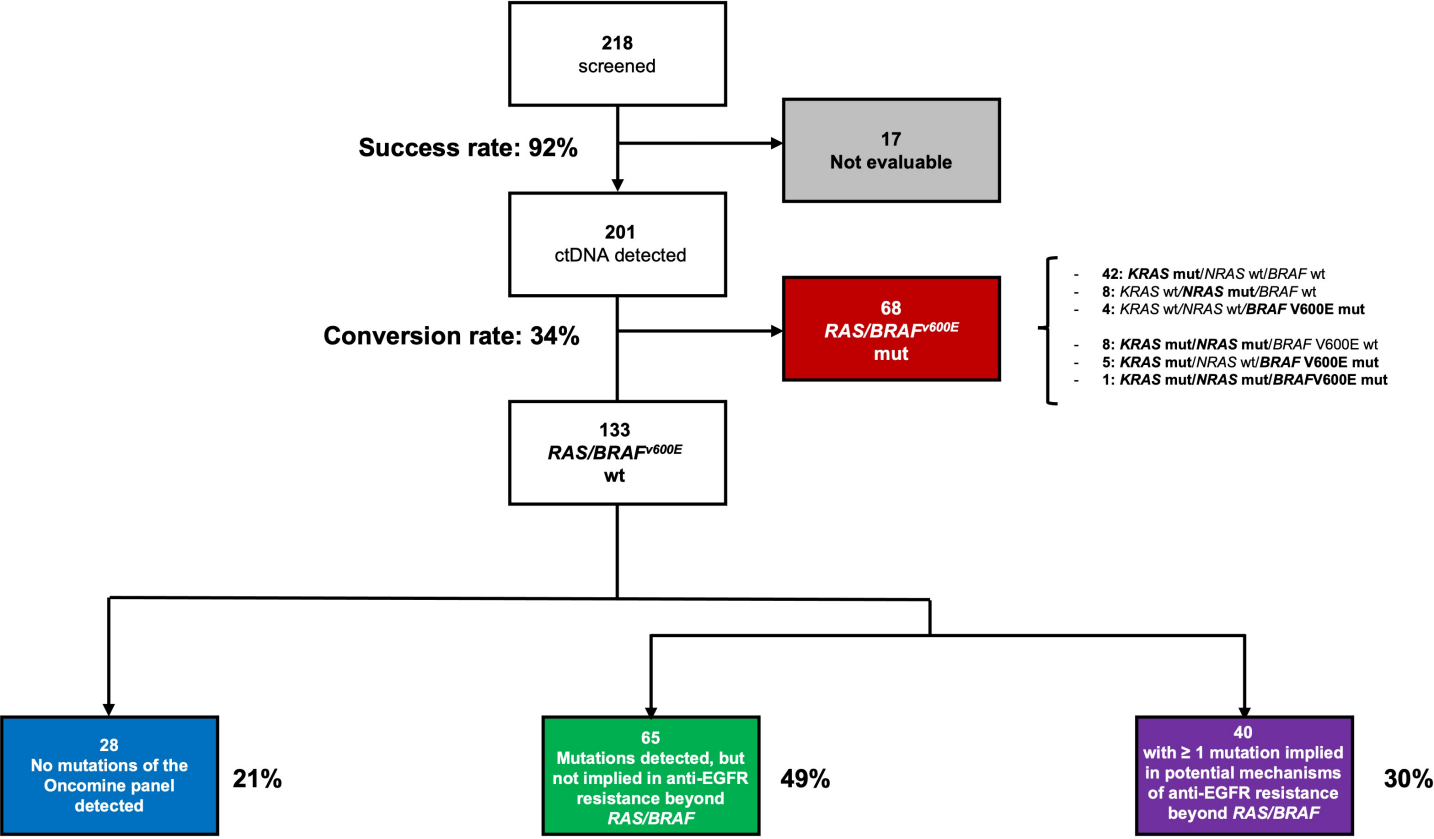
ctDNA screening results according to successful sequencing, RAS/BRAF genotyping and other mutations in the Oncomine panel.

Between December 2020 and January 2023, 218 patients met the eligibility criteria for screening, and ctDNA genotyping was successful in 201 (92%) cases, with a median LOD of 0.11%. *KRAS*, *NRAS*, or *BRAF*V600E mutations were found in 68 (34%) patients. Co-mutations were reported in 14 cases, including 8 cases of *KRAS/NRAS* mutations (11%), 5 cases of *KRAS/BRAF*V600E mutations (7%), and 1 case of *KRAS/NRAS/BRAF*V600E mutations (1%). A total of 133 patients were *RAS* and *BRAF*V600E wt and were thus eligible for randomization in the PARERE trial (**Figure 1**).

**Figure 2:**
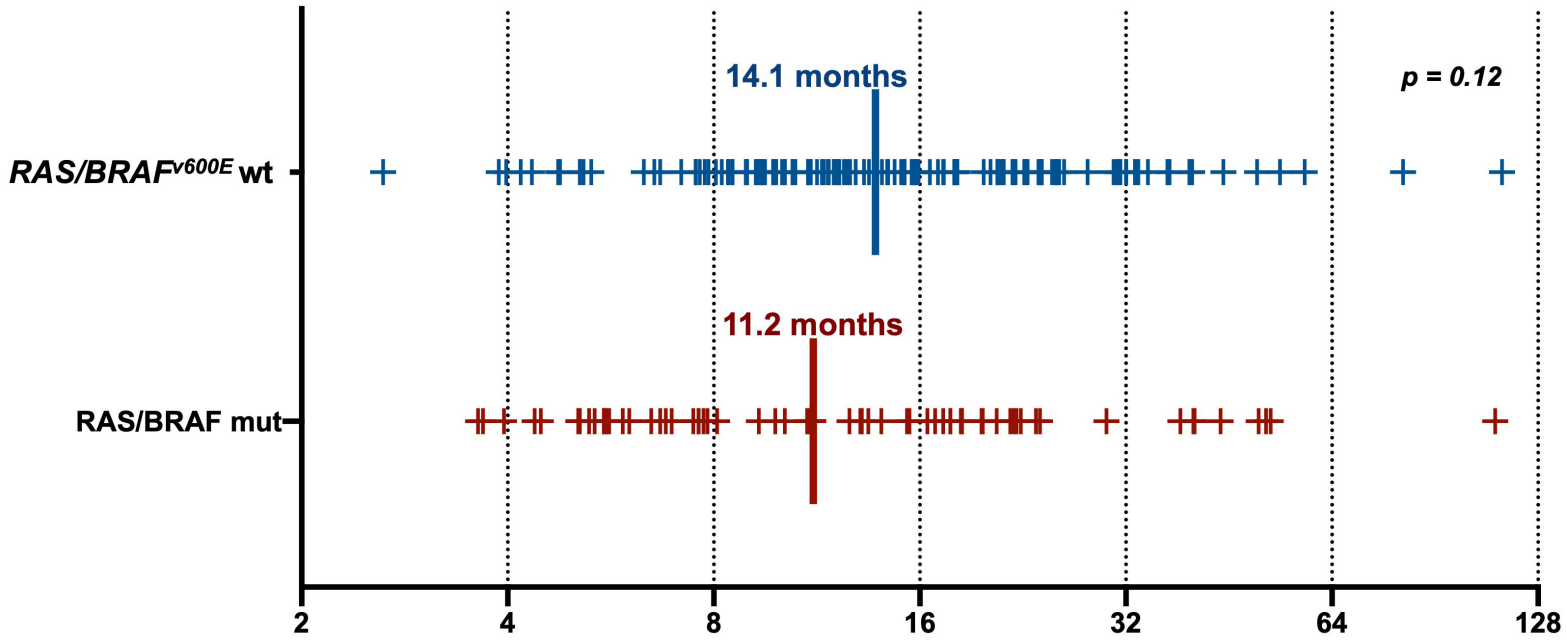
Anti-EGFR free interval in ctDNA RAS/BRAFV600E wild-type and mut patients.

Clinical characteristics of patients with *RAS/BRAF*V600E wt versus *RAS/BRAF*V600E mutated ctDNA are summarized in **Table 1**. None of them was predictive of ctDNA status at screening for anti-EGFR retreatment, except for a higher percentage of patients with liver-limited disease at the time of the previous anti-EGFR exposure in the *RAS/BRAF* wt ctDNA group (41% versus 24%, *p* = 0.02). Notably, the interval between the last anti-EGFR administration and the time of screening was not statistically different between the *RAS/BRAF*V600E wt and the *RAS/BRAF*V600E mutated cohorts (14.1 versus 11.2 months, *p* = 0.12) (**Figure 2**).

**Table 1:** Patients’ characteristics according to ctDNA molecular status by PARERE screening.

Characteristics		RAS/BRAFV600E wt	RAS/BRAFV600E mut	p
		*n* = 133 (%)	*n* = 68 (%)	
Sex	Male	74 (56)	38 (56)	0.97
	Female	59 (44)	30 (44)	
Primary tumor location	Left colon and rectum	120 (90)	63 (93)	0.57
	Right colon	13 (10)	5 (7)	
Surgery on primary tumor	Yes	107 (81)	51 (75)	0.37
	No	26 (19)	17 (25)	
Mucinous histology	Yes	8 (6)	4 (6)	1.00
	No	104 (78)	61 (90)	
	NA	21 (16)	3 (4)	
MSI status	pMMR or MSS/MSI-low	131 (99)	68 (100)	0.55
	dMMR or MSI-high	2 (1)	0 (0)	
Time to metastases	>3 months (metachronous)	31 (23)	19 (28)	0.49
	≤3 months (synchronous)	101 (76)	49 (72)	
	NA	1 (1)	0 (0)	
Liver-limited disease at the start of previous anti-EGFR-based treatment	Yes	54 (41)	16 (24)	**0.02**
	No	79 (59)	52 (76)	
Peritoneal metastases at the time of LB screening	Yes	27 (20)	13 (19)	0.84
	No	106 (80)	55 (81)	
Number of metastatic sites at the time of LB screening	1	19 (14)	8 (12)	0.21
	2	48 (36)	23 (34)	
	3	50 (38)	21 (31)	
	> 3	16 (12)	16 (23)	
Previous anti-EGFR-based treatment regimen	FOLFIRI + anti-EGFR	29 (22)	16 (23)	0.73
	FOLFOX/XELOX + anti-EGFR	78 (59)	43 (63)	
	FOLFOXIRI + anti-EGFR	19 (14)	7 (10)	
	Anti-EGFR ± monochemotherapy	7 (5)	2 (3)	
Previous anti-EGFR-based treatment response	CR/PR	111 (84)	60 (88)	0.37
	SD	22 (16)	8 (12)	
mPFS to previous anti-EGFR exposure	Median, months	13.1	14.3	0.95
	(95% CI)	(12.3–63.6)	(12.0–67.1)	
Time interval between last anti-EGFR administration and disease progression	>3 months	62 (47)	39 (57)	0.15
	≤3 months	71 (53)	29 (43)	
Time interval between last anti-EGFR exposure and LB screening	Median, months	14.1	11.2	0.12
	(95% CI)	(12.3–15.7)	(9.9–15.5)	
Lines of treatment between last anti-EGFR exposure and LB screening	None	1 (1)	1 (1)	0.75
	1	98 (74)	50 (74)	
	2	26 (20)	14 (21)	
	3	6 (4)	1 (1)	
	4	2 (1)	2 (3)	

MSI, microsatellite instability; anti-EGFR, anti-epidermal growth factor receptor monoclonal antibodies; CI, confidence interval; CR, complete response; LB, liquid biopsy; NA, not available; PFS, progression-free survival; PR, partial response, SD, stable disease; ctDNA, circulating tumor DNA; pMMR, proficient mismatch repair; dMMR, deficient mismatch repair. Bolded value corresponds to a statically significant p-value as compared with the others (not statistically significant).

The LOD was superimposable in patients with *RAS/BRAF*V600E wt and *RAS/BRAF*V600E mutant clones (0.12% versus 0.10%, respectively, *p* = 0.46) (**Supplementary Figure 1**). The distribution of variant allele fraction (VAF) among *KRAS*, *NRAS*, and *BRAF* mutant tumors was not homogeneous (*p* = 0.01), with *BRAF* mutant clones showing a lower VAF compared to *KRAS* (0.19% versus 0.52%, *p* = 0.009) and *NRAS* mutant clones (0.19 versus 0.62%, *p* = 0.07) (**Supplementary Figure 2**). Among *KRAS* alterations, the most frequent driver of secondary resistance to anti-EGFRs was *KRAS*Q61H mutation (31%), followed by *KRAS*G12D (21%) and *KRAS*G12A (16%). The druggable *KRAS*G12C mutation was found in 3% of patients with *KRAS* mutant ctDNA (**Supplementary Figure 3**).

**Figure 3:**
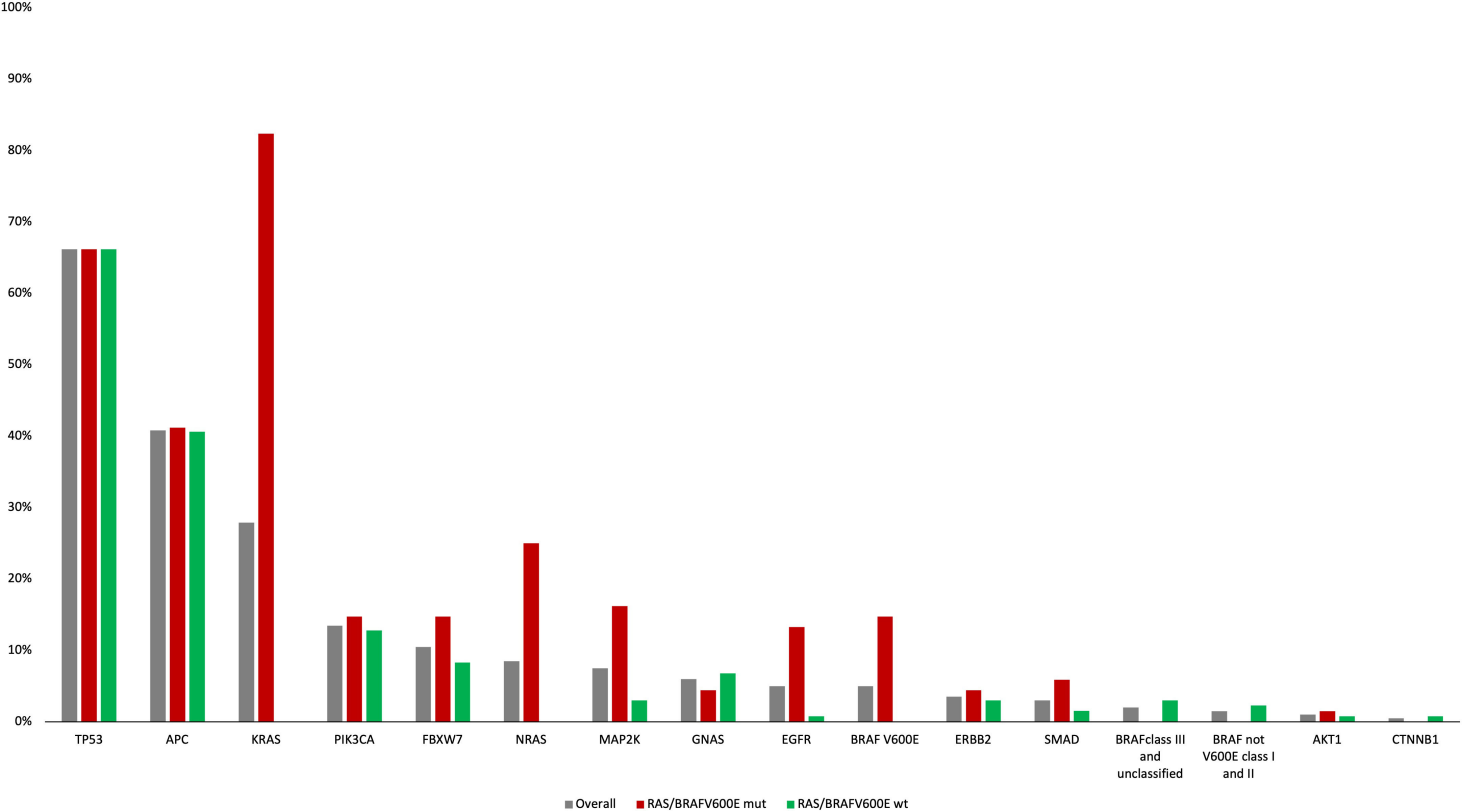
Frequency of genomic alterations according to the Oncomine panel in the overall, RAS/BRAFV600E mut and RAS/BRAFV600E wt populations.

**Figure 4:**
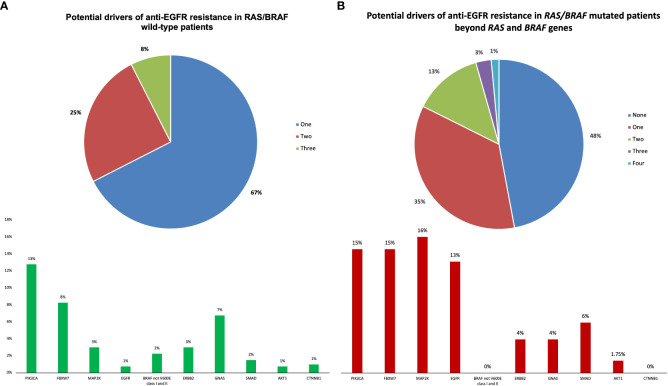
Potential drivers of anti-EGFR resistance beyond RAS/BRAF genes in RAS/BRAF wild-type and mutated patients.

Among *RAS/BRAF*V600E wt patients, 28 (21%) did not harbor any mutation included in the Oncomine™ panel. Among the others, mutations in founder genes such as *TP53* and *APC* were detected in 88 (66%) and 53 (40%) patients, respectively, with frequent co-mutations. Most importantly, 40 out of 133 *RAS/BRA*FV600E wt patients (30%) had a mutation in at least another gene potentially implied in anti-EGFR resistance—including class I and II non-*BRAF*V600E mutations (**Figure 3**). Notably, 27 patients (67%) harbored one single potential driver of resistance, with co-mutations in two or three genes occurring in 10 (25%) and 3 (8%) cases, respectively. In detail, mutations in *PIK3CA*, *FBXW7*, *GNAS*, *MAP2K1*, *ERBB2*, *BRAF* (class I and II non-*BRAF*V600E), *SMAD*, *EGFR*, *AKT1*, and *CTNNB1* occurred in 13%, 8%, 7%, 3%, 2%, 2%, 1%, 1%, 1%, and 1% of patients, respectively. Of 68 patients, 36 (53%) with *RAS* and/or *BRAF*V600E mutant clones showed the co-presence of other drivers of resistance as well (**Figure 4A**). Overall, one, two, three, and four other potential drivers of resistance to anti-EGFRs were found in 67%, 25%, 6%, and 1% of patients, respectively. The relative frequency of these co-mutations was different compared to that of RAS/BRAFV600E wt patients, with a higher frequency of *MAP2K1* (16%), *FBXW7* (15%), *EGFR* (13%), and *SMAD* (6%) mutations (**Figure 4B**).

The median VAF of altered genes other than *RAS* and *BRAF*V600E driving potential resistance to anti-EGFRs was subclonal and numerically higher in the *RAS/BRAF*V600E wt group compared to the *RAS/BRAF*V600E mutant (0.56% versus 0.23%, *p* = 0.22) (**Supplementary Figure 4**).

## 4 Discussion

A growing number of therapeutic options have recently become available in the chemorefractory landscape of mCRC patients, and others are under investigation, including anti-EGFR retreatment in patients with *RAS/BRAF* wt tumors (1, 10, 12–14). In this regard, the most convincing evidence has been collected in patients selected according to the analysis of ctDNA by means of high-throughput sequencing technologies (i.e., digital droplet PCR) aimed at revealing the lowest fraction of resistant clones in the blood while limiting the analysis to a restricted number of genes (i.e., *RAS*, *BRAF*, and *EGFR*) (1, 4, 5). In patients with *RAS*, *BRAF*, and *EGFR* wt ctDNA, according to digital droplet PCR (ddPCR), the anti-EGFR retreatment provided a 30% ORR and 4-month PFS (1, 4, 5). The positioning of anti-EGFR retreatment in the current landscape of therapeutic options for chemorefractory mCRC patients will be likely clarified by evidence from randomized trials. This is the objective of the PARERE study: to adopt an NGS technique as a screening tool and to clarify in a *post-hoc* analysis the impact of other alterations of acquired resistance.

Our series is the largest prospective cohort (*n* = 202) of patients candidates for anti-EGFR retreatment undergoing molecular screening in LB. The *RAS/BRAF*V600E conversion rate of 34% reported here mirrors previous data from the CHRONOS trial (29%) obtained with a digital droplet PCR assay (1), suggesting that the flexible LOD of the NGS approach used in the PARERE study still provides the advantage of a massively parallel sequencing while preserving an adequate sensitivity to detect RAS/BRAF mutant clones (1). *KRAS*Q61H and *KRAS*G12A hotspot mutations, respectively accounting for 35% and 16% of identified *KRAS* mutations, were much more frequent than in the chemo-naïve setting where their incidence is approximately 1.5% and 4%, respectively, among *KRAS* mutant tumors (15–17). Our data corroborate previous findings by Woolston et al. and highlight that *KRAS* mutations selected under the pressure of EGFR blockade follow different mechanisms of molecular adaptation than those selected in earlier stages of the disease (18). From a biochemical standpoint, *KRAS* codon 61 mutations yield higher *in vitro* KRAS-GTP levels as compared to codon 12 and 13 mutations and share with *KRAS* G12A a lower intrinsic GTPase activity and a stronger affinity for *RAF* gene, as compared to mutations in other codons (19, 20). These considerations may be relevant in the current and future research efforts toward mutation-specific and pan-KRAS inhibitors (21, 22).

Notably, no clinical characteristics were reliable in predicting *RAS/BRAF*V600E status (1). Numerically longer anti-EGFR-free intervals and time from the last anti-EGFR administration to first-line disease progression were observed in the group with *RAS/BRAF*V600E wt ctDNA. Translational data from the randomized FIRE4 trial showed a significant correlation between the duration of the exposure to first-line anti-EGFRs and the occurrence of *RAS* mutations, though in the absence of any overall survival (OS) difference between the groups of patients acquiring or not *RAS* mutations in ctDNA (23).

We managed to detect rare subclonal alterations potentially implied in anti-EGFR resistance in the 30% of *RAS/BRAF*V600E wt patients, with co-mutations occurring in roughly one-third of them, providing further evidence that co-evolution of different subclones is a common mechanism of escape to targeted therapy, as recently observed in patients with RAS wt tumors treated with an anti-EGFR and then enrolled in the CO.26 trial (24) and in *BRAF*V600E mutant mCRC patients treated with encorafenib plus cetuximab in the BEACON trial (25). Among *RAS* and *BRAF* wt ctDNA patients, potential drivers of anti-EGFR resistance were found in 28% of subjects, mostly including *PIK3CA*, *FBXW7*, and *GNAS* mutations (28%), whereas *AKT*, *EGFR*, *SMAD*, and *CTNNB1* mutations were rare (4%). Remarkably, patients with *RAS/BRAF*V600E mutations in their ctDNA showed a different genomic pattern, with redundant activation of the MAPK pathway through *EGFR* and *MAP2K1* mutations in 29% of patients.

Our work suffers from several limitations. First, blood samples were collected and analyzed only at the time of screening for the PARERE study and not at the diagnosis of mCRC, thus not allowing for full exclusion of *RAS/BRAF* mutations that could be detected in ctDNA before the first-line anti-EGFR-based therapy, though in the absence of mutations in tissue DNA, as described in up to 10% of *RAS/BRAF*V600E wt mCRC patients (26). Second, the Oncomine™ panel does not allow the identification of molecular alterations other than SNVs (i.e., amplifications or rearrangements) and covers only 14 genes; other drivers of anti-EGFR resistance may have been missed at screening. Third, only outcome data from the PARERE trial will clarify the impact of these alterations on the clinical activity of the anti-EGFR retreatment.

In conclusion, preliminary molecular findings from the PARERE study corroborate the need to use LB as a screening tool before offering anti-EGFR retreatment, considering the lack of reliability of potential clinical surrogates of EGFR dependency. The subclonality of molecular alterations identified in these patients makes quite challenging the idea of exploiting these events as efficacious targets for subsequent tailored treatments.

## Data availability statement

The original contributions presented in the study are publicly available. This data can be found here: https://www.ncbi.nlm.nih.gov/sra/PRJNA1064885.

## Ethics statement

The studies involving humans were approved by Comitato Etico Regione Toscana - Area Vasta Nord Ovest (CEAVNO) - Codice OsSC: OCE000000056 - ORG ID: ORG-100032439. The studies were conducted in accordance with the local legislation and institutional requirements. The participants provided their written informed consent to participate in this study.

## Author contributions

MMG: Conceptualization, Data curation, Formal Analysis, Investigation, Methodology, Writing – original draft, Writing – review & editing, Supervision, Visualization, Validation. GV: Data curation, Formal Analysis, Investigation, Writing – original draft, Validation. MG: Data curation, Writing – review & editing, Investigation. PC: Data curation, Formal Analysis, Investigation, Writing – original draft, Writing – review & editing, Validation. IC: Data curation, Writing – review & editing, Investigation. ET: Data curation, Writing – review & editing, Investigation. EC: Data curation, Writing – review & editing, Investigation. ABu: Data curation, Writing – review & editing, Investigation. FP: Data curation, Writing – review & editing, Funding acquisition, Project administration, Resources, Supervision, Validation. VP: Data curation, Writing – review & editing, Validation. ABo: Data curation, Writing – review & editing, Validation. FS: Data curation, Writing – review & editing, Validation. MB: Data curation, Writing – review & editing, Validation. PM: Data curation, Writing – review & editing, Validation. SL: Data curation, Writing – review & editing, Funding acquisition, Project administration, Resources, Supervision, Validation. VC: Data curation, Writing – review & editing, Validation. BB: Data curation, Writing – review & editing, Validation. MC: Data curation, Writing – review & editing, Validation. MR: Writing – review & editing, Investigation, Supervision, Validation. GF: Writing – review & editing, Investigation, Supervision, Validation. DR: Data curation, Formal Analysis, Writing – review & editing, Conceptualization, Investigation, Supervision, Validation, Writing – original draft. CC: Conceptualization, Data curation, Investigation, Methodology, Supervision, Visualization, Writing – original draft, Writing – review & editing, Formal Analysis, Funding acquisition, Project administration, Resources, Validation.
