# Supplementary figures and images for: Molecular screening with liquid biopsy for anti-EGFR retreatment in metastatic colorectal cancer: preliminary data from the randomized phase 2 PARERE trial
Source: Front Oncol. 2024 Feb 9;13:1307545. doi: 10.3389/fonc.2023.1307545 (PMC10889120; doi:10.3389/fonc.2023.1307545)

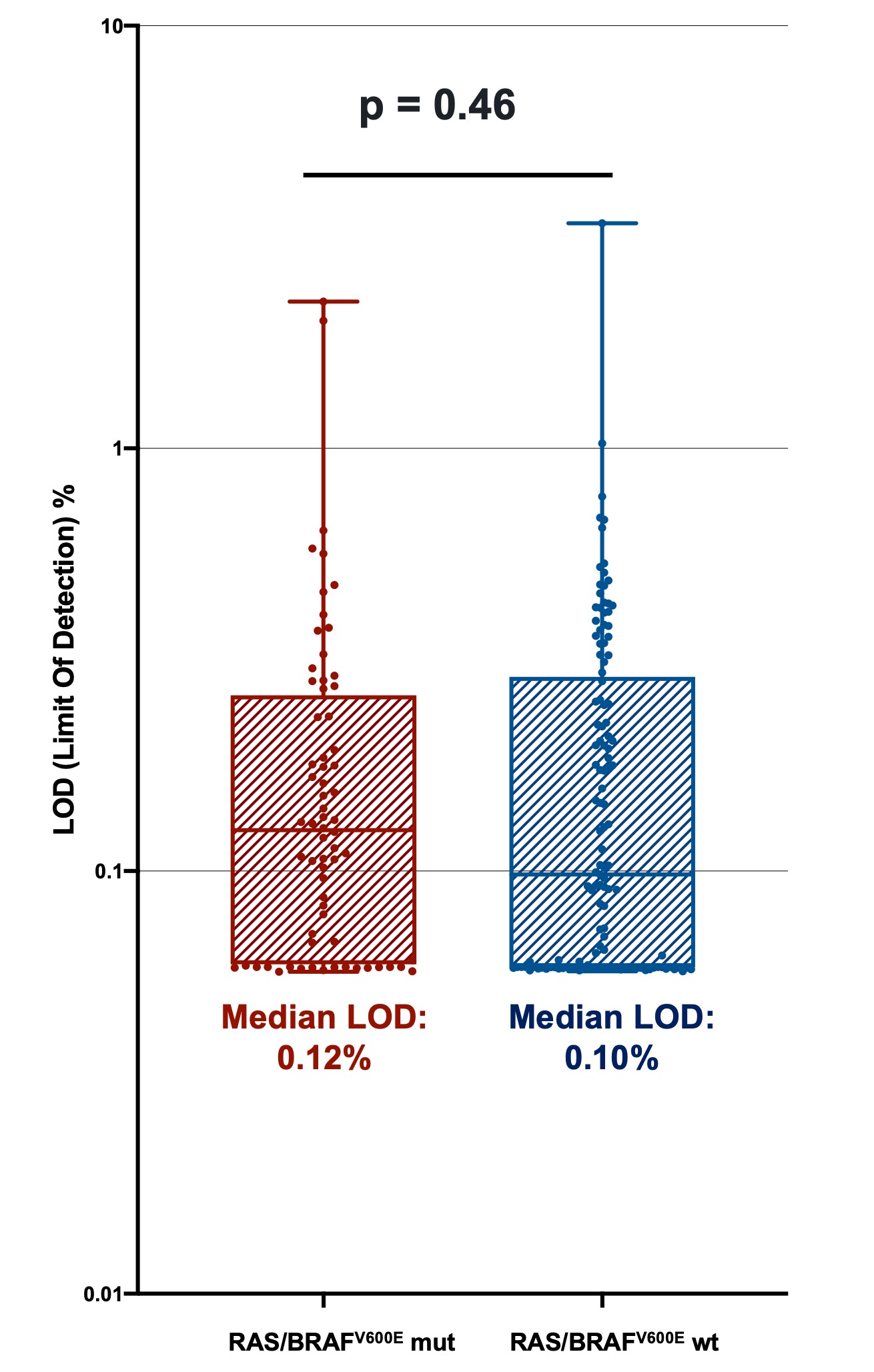

Supplement: Supplementary file 1 [file Image_1.jpeg]

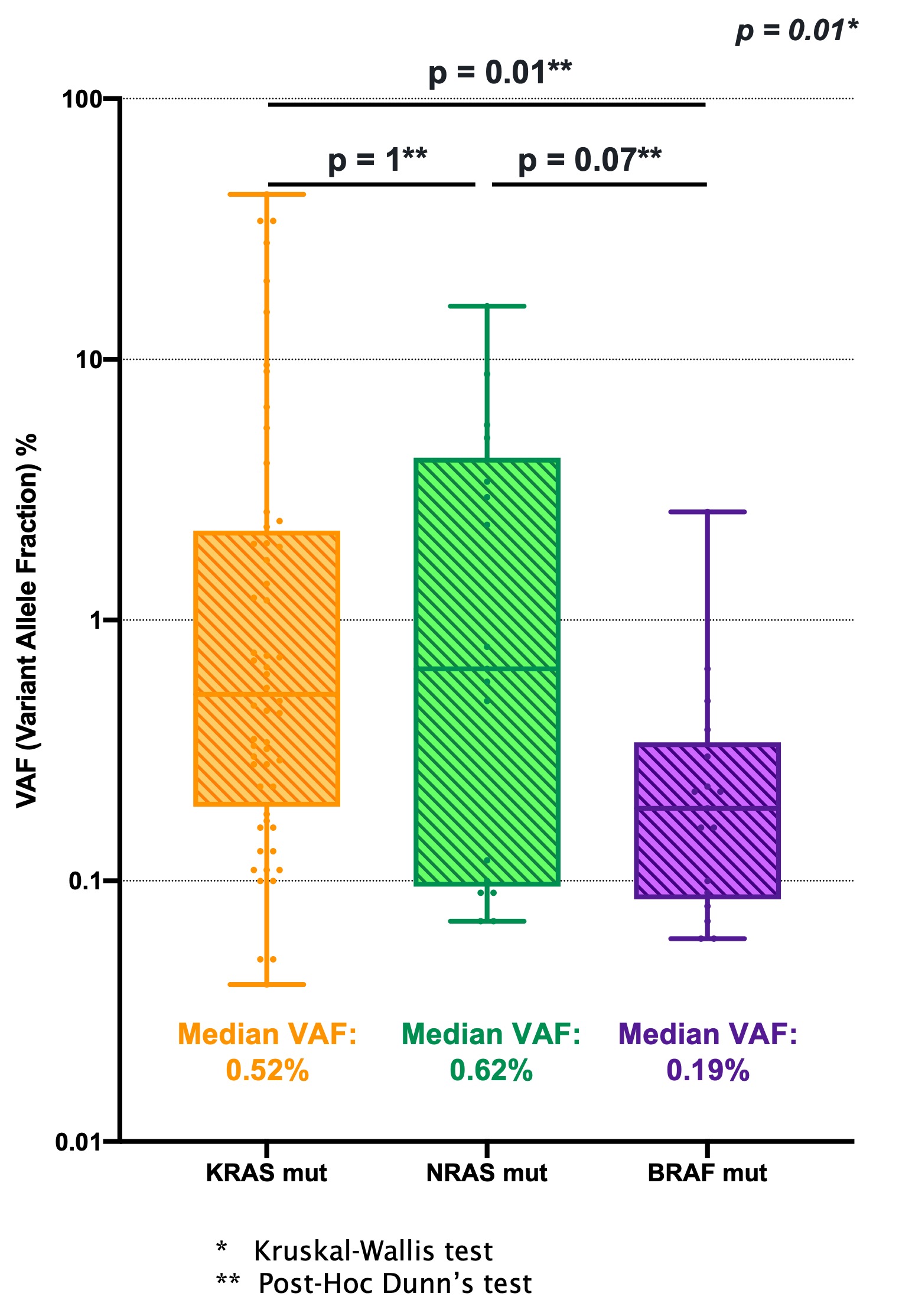

Supplement: Supplementary file 2 [file Image_2.jpeg]

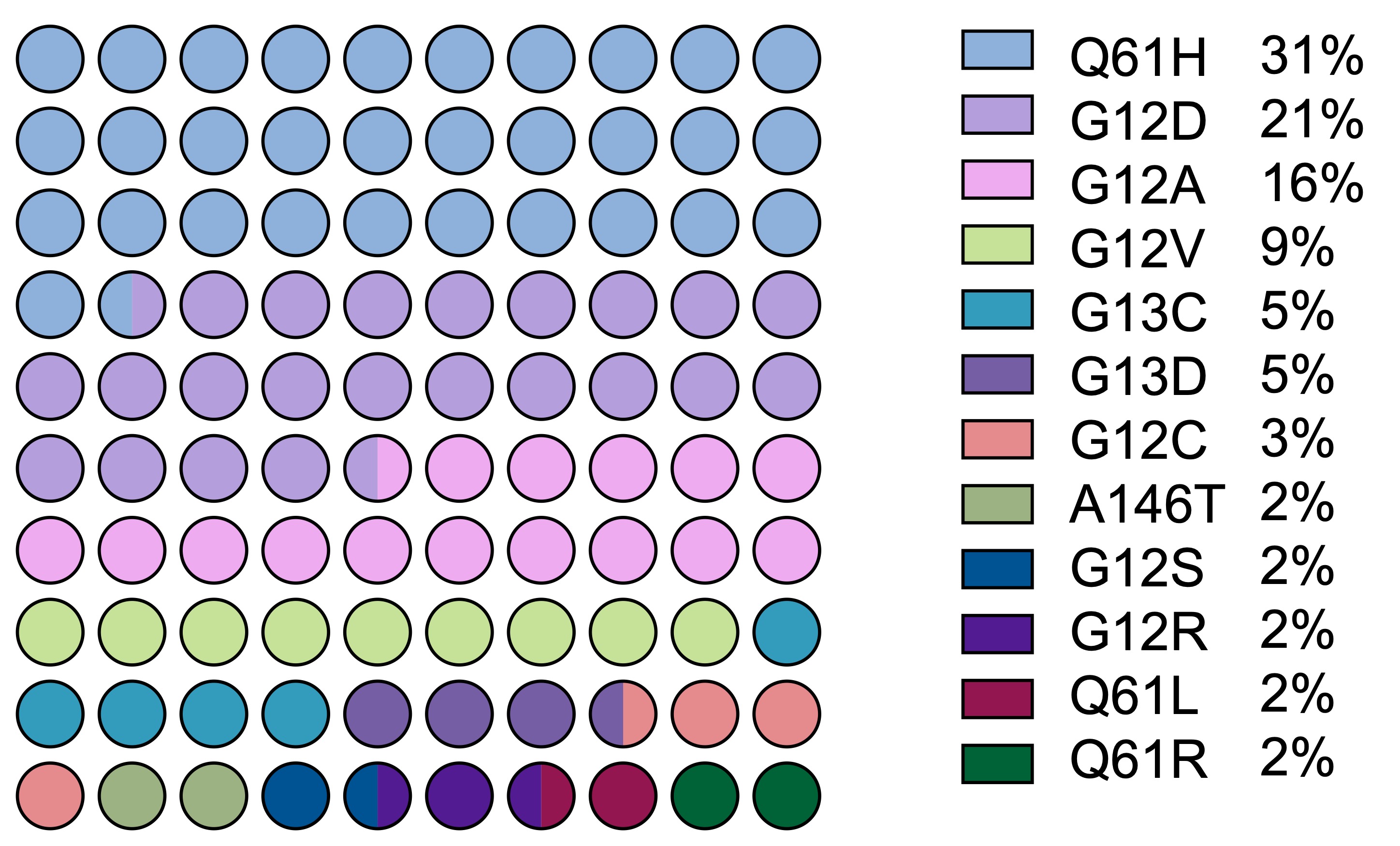

Supplement: Supplementary file 3 [file Image_3.jpeg]

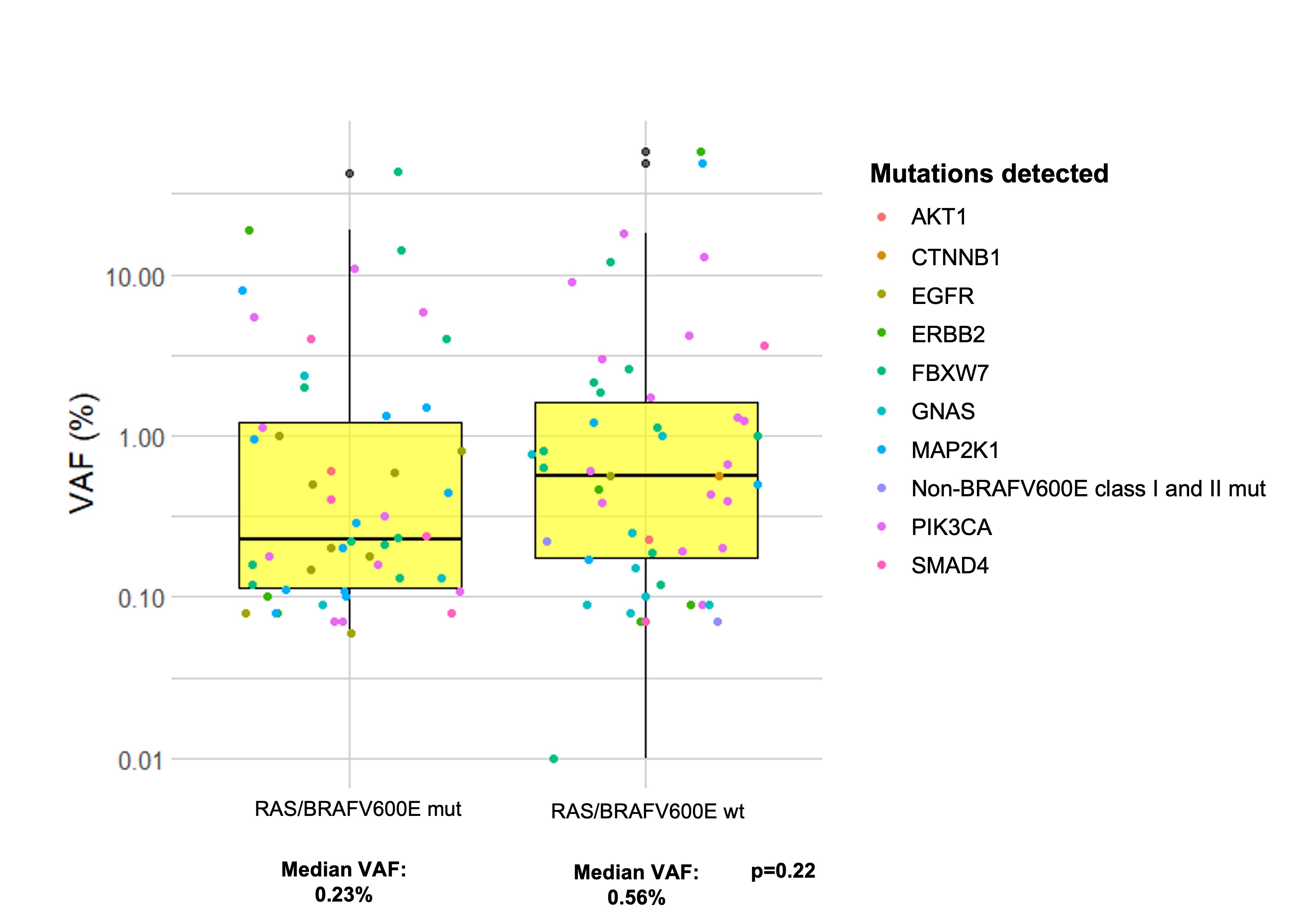

Supplement: Supplementary file 4 [file Image_4.jpeg]
